# Role of Vonoprazan in *Helicobacter pylori* Eradication Therapy in Japan

**DOI:** 10.3389/fphar.2018.01560

**Published:** 2019-01-15

**Authors:** Mitsushige Sugimoto, Yoshio Yamaoka

**Affiliations:** ^1^Division of Digestive Endoscopy, Shiga University of Medical Science Hospital, Otsu, Japan; ^2^Department of Environmental and Preventive Medicine, Faculty of Medicine, Oita University, Yufu, Japan

**Keywords:** *Helicobacter pylori*, eradication therapy, vonoprazan, intragastric pH, clarithromycin

## Abstract

Complete eradication of *Helicobacter pylori* is important for preventing the development of gastric cancer. The outcome of *H. pylori* eradication therapy is mainly dependent on bacterial susceptibility to antimicrobial agents and potent neutralization of intragastric pH across 24 h, especially when using acid-sensitive antimicrobial agents such as clarithromycin (CLR), amoxicillin and sitafloxacin. However, conventional regimens comprising twice-daily doses (bid) of proton pump inhibitors (PPIs) are generally insufficient for maintaining the required gastric acid secretion for 24 h for successful eradication in all *H. pylori*-positive patients. Further, the increasing prevalence of CLR-resistant strains with each year has led to a decrease in eradication rates of first-line PPI- and CLR-containing therapies in developed countries, including Japan. In 2015, the potassium-competitive acid blocker vonoprazan (VPZ) became clinically available in Japan. VPZ competitively inhibits H^+^/K^+^-ATPase activity more potently than PPIs (e.g., omeprazole, lansoprazole, rabeprazole, pantoprazole, and esomeprazole). Therefore, a VPZ-containing *H. pylori* eradication regimen is expected to increase the eradication rate compared with conventional regimens containing a standard dose of PPI. In fact, a recent meta-analysis that investigated the efficacy of first-line eradication therapy showed that a VPZ-containing regimen achieved a higher eradication rate than a PPI-containing regimen. While the Maastricht V/Florence Consensus Report recommends selecting a bismuth or non-bismuth quadruple therapy and concomitant therapy for patients living in areas with high prevalence of CLR resistance, a VPZ-containing regimen demonstrates effectiveness for patients infected with CLR-resistant strains and patients living in areas where the prevalence of CLR-resistant strains is >15%. As a next step, studies are needed to determine the factors affecting the clinical outcome of VPZ-containing therapy and optimal VPZ-containing alternative regimens for tailored treatments. In this review, we summarize the advantages and disadvantages of VPZ in *H. pylori* eradication therapy.

## Introduction

Rapid and potent acid inhibition after treatment with acid-inhibitory drugs (i.e., proton pump inhibitor [PPI]) is necessary for curing acid-peptic disorders. Treatments that neutralize intragastric pH levels are associated with improved cure rates for peptic ulcers (Barer et al., 1983), gastroesophageal reflux disease (Bell et al., 1992), non-erosive reflux diseases, aspirin-induced and non-steroidal anti-inflammatory drug-induced gastroduodenal mucosal injury (Sugimoto et al., 2012a), and *Helicobacter pylori* infection (Labenz et al., 1995; Sugimoto et al., 2007; Yang et al., 2011). Therefore, an understanding of methods to suppress gastric acid secretion is important in the treatment of acid-peptic disorders.

The new, potent acid-inhibitory drug vonoprazan (VPZ) recently became clinically available in Japan. VPZ competitively inhibits the binding of potassium ions to H^+^/K^+^-ATPase in gastric parietal cells more potently than PPIs (Parsons and Keeling, 2005). VPZ also has two pharmacological advantages over PPIs: it does not require pharmacological activation by gastric acid to inhibit acid secretion, and has a longer half-life (t1/2) due to its slow dissociation kinetics from H^+^/K^+^-ATPase (Sugimoto et al., 2004; Scott et al., 2015). While PPIs typically require more than 75–100 h to exert a maximal gastric acid inhibitory effect (Saitoh et al., 2002; Sugimoto et al., 2006), VPZ produces rapid, strong and long-lasting gastric acid inhibition after administration of the first tablet in a dose-dependent manner (Jenkins et al., 2015; Sakurai et al., 2015). At steady state on Day 7, a once daily dose (oid) of VPZ 40 mg displayed sustained and potent acid inhibition throughout a 24-h period (Jenkins et al., 2015). Moreover, a twice daily dose (bid) of VPZ 20 mg, the standard dosage for *H. pylori* eradication therapy, maintained gastric acid inhibition throughout the 24 h: the pH > 4 and >5 holding time ratio (HTR) was 100 and 99%, respectively, even in *H. pylori*-negative subjects (Kagami et al., 2016). Therefore, VPZ may be an effective first-line acid-inhibitory drug for patients with acid-peptic disorders.

In Japan, *H. pylori* eradication therapies are currently limited to regimens comprising an acid-inhibitory drug such as a PPI or VPZ at a standard dose bid, amoxicillin (AMX) 750 mg bid, and clarithromycin (CLR) 200 mg or 400 mg bid for 7 days as a first-line eradication regimen; and PPI or VPZ bid, AMX 750 mg bid, and metronidazole (MNZ) 250 mg bid for 7 days as a second-line eradication regimen. Unfortunately, the frequent use of CLR in general clinical situations has led to an increase in the prevalence of CLR-resistant *H. pylori* strains in Japan (more than 30%) (Asaka et al., 2001; Murakami et al., 2002), prompting the need for alternative regimens. Because potent neutralization of pH is associated with better outcomes of *H. pylori* eradication therapy, as it is for other acid-peptic disorders (Labenz et al., 1995; Sugimoto et al., 2007; Yang et al., 2011), VPZ may dramatically improve the decreasing *H. pylori* eradication rate in Japan.

Here, we discuss the impact of VPZ in *H. pylori* eradication therapy, as well as some of its disadvantages. We first discuss the general factors influencing the cure rate for *H. pylori* infection due to *H. pylori* eradication therapy and association with the outcome of eradication and importance of inhibiting acid secretion. We subsequently discuss the efficacy of VPZ-containing eradication therapy of first-, second- and third-line treatments, and in patients with penicillin allergies.

## Possible Factors Contributing to the Outcome of Eradication Therapy for *H. pylori* Infection

The cure rate for *H. pylori* infection is affected by several possible factors, as below: antibiotic susceptibility (e.g., CLR, AMX, MNZ and levofloxacin) (Asaka et al., 2001; Furuta et al., 2001; Murakami et al., 2002), insufficient acid inhibition during eradication therapy (e.g., *CYP2C19* and *CYP3A4/5* genotype, dose of drug, treatment schedule and type of acid-inhibitory drug) (Furuta et al., 2001; Sugimoto et al., 2007; Sugimoto and Yamaoka, 2009), the environment (e.g., smoking), and poor adherence to medication and *H. pylori* strain with low virulence activity (e.g., *cagA*-negative strains, *vacA* s2 genotype and *dupA*-negative strains) (Sugimoto and Yamaoka, 2009; Shiota et al., 2012; Table 1). Although much attention is focused on the relationship between resistant strains of *H. pylori* and the success or failure of eradication therapy, potent acid inhibition throughout the 24 h during eradication therapy has become re-recognized as an important outcome of eradication therapy. In fact, the Maastricht V/Florence Consensus Report states that “the use of high dose PPI bid increases the efficacy of triple therapy. Level of evidence: low, and Grade of recommendation: weak” (Malfertheiner et al., 2017).

**Table 1:** Major potential factors influencing the outcome of *H. pylori* eradication therapy.

Category	Factor		
Antibiotics	Resistance to antibiotics	Clarithromycin	A2142G, A2142C, and A2143G mutations in the *23S* rRNA gene
		Metronidazole	frxA (hp0642), rdxA (hp0954), and rpsU (hp0562) mutations
		Levofloxacin	C261A/G, C271A/T, and A272G mutations in *gyrA*
		Amoxicillin	Multiple point mutations in *pbp1* gene
Acid inhibition	Insufficient acid inhibition	CYP2C19 type (PPI)	Extensive metabolizer (^∗^1/^∗^1 type)
		*CYP2C19*^∗^17 (PPI)	^∗^17 carrier
		*ABCB1* 3435 (PPI)	C/C genotype (Caucasian)
		CYP3A5 (VPZ)	^∗^1 carrier
		*IL-1B*-511	C/C genotype
		*IL-1B*-31	T/T genotype
		Time of dosing	Low frequency (i.e., oid)
		Drug dose	Insufficient dose
*H. pylori* phenotype	*H. pylori* virulence factors	*cagA* status	Negative
		*vacA* genotype	s2 type
		*dupA* status	Negative
	Volume		Much
Environment	Smoking		Many
	Adherence		Insufficient

*ABCB1, multidrug resistance protein-1; CYP2C19, cytochrome P450 2C19; CYP3A5, cytochrome P450 3A5; IL, interleukin.*

Control of pH using acid-inhibitory drugs depends on the type of acid-inhibitory drug, dosage (dose and dosing time), combination of drugs (e.g., any of PPI, VPZ and histamine 2 receptor antagonist) and polymorphisms in drug-metabolizing enzyme genes (e.g., cytochrome P450 2C19 (CYP2C19), CYP3A4, and CYP3A5) and polymorphisms in drug transporter genes (e.g., multidrug resistance protein-1 [ABCB1]) that effect the pH during treatment (Table 1; Furuta et al., 1999; Shirai et al., 2001, 2002; Sugimoto et al., 2004, 2005, 2012b; Kodaira et al., 2009; Kagami et al., 2016).

## Importance of Gastric Acid Inhibition in *H. pylori* Eradication Therapy

*Helicobacter pylori* can survive a periplasmic pH of 4.0–8.0 in the gastric mucosa (Scott et al., 1998). When the bacterial urease activity of *H. pylori* raises the intragastric pH to 4.0–6.0, *H. pylori* survives into the gastric mucosa but does not divide (Scott et al., 1998). Therefore, the consistent and potent action of acid-inhibitory drugs also enables *H. pylori* to grow and become more sensitive to antimicrobial agents against *H. pylori* (Scott et al., 1998). In addition, potent acid inhibition during 24-h increases the stability and bioavailability of acid-sensitive antimicrobial agents by preventing their degradation. Further, PPI and vonoprazan increase gastric mucosal antimicrobial agents concentration (Grayson et al., 1989; Goddard et al., 1996; Scott et al., 1998). Raising the intragastric pH from 3.5 to 5.5 is shown to increase the *in vitro* antimicrobial efficacy of AMX more than 10-fold (Grayson et al., 1989). The activity of CLR against *H. pylori* is higher at intragastric pH 7.4 than at intragastric pH 5.0, and activity is intermediate at pH 6.8 (Heifets et al., 1992). Recently, the Maastricht V/Florence Consensus Report recommended first-line eradication therapy using a CLR-containing regimen with PPI/AMX or PPIMNZ and an alternative eradication treatment using bismuth-containing quadruple treatment (PPI/bismuth/MNZ/tetracycline) in areas where prevalence of CLR-resistant strains is low (Level of evidence: high, Grade of recommendation: strong), and bismuth or non-bismuth quadruple treatment and concomitant (PPI/AMX/CLR/nitroimidazole) therapies in areas of high (>15%) CLR resistance (Level of evidence: low, Grade of recommendation: strong) (Malfertheiner et al., 2017). These recommendations indicate that there are numerous opportunities to use acid-sensitive antimicrobial agents around the world, and underlines the need to monitor inhibition of gastric acid secretion in eradication treatment.

However, the question of how strongly gastric acid secretion should be inhibited remains. Previously, we showed, using PPI/AMX/CLR triple therapy, a standard eradication regimen around the world, that the median 24-h pH for successful eradication was higher (6.4) and the median pH < 4 HTR (0.5%) was shorter than that for failed eradication (pH 5.2 and pH < 4 HTR 26.7%) (Sugimoto et al., 2007). Therefore, the degree and duration of acid inhibition during eradication therapy are related to the cure rate of *H. pylori*, and we concluded that intragastric pH > 4 should be maintained for 24 h and that the 24-h intragastric pH should be higher than 6.0 (Sugimoto et al., 2007).

Unfortunately, treatment with PPI at standard dose bid does not maintain pH values at higher than 4.0 for long enough to accept the above criteria in all patients receiving eradication therapy (Sugimoto et al., 2004). Therefore, it is necessary to identify the factors that affect acid secretion and to determine the optimal drug therapy to enable the inhibition of acid secretion across 24 h. Because VPZ 20 mg bid inhibits acid secretion across 24 h (pH ≥ 4 HTR is 100%), (Kagami et al., 2016) VPZ is an effective acid-inhibitory drug in eradication therapies for *H. pylori* infection. There is no report to investigate direct association with advantage of vonoprazan use and acid inhibition during vonoprazan-containing eradication therapy for better outcome of *H. pylori* eradication therapy.

## First-Line Vonoprazan-Containing Eradication Therapy

### Study Selection

In this review, we searched for all of relevant studies published up until September 2018 that examined the efficacy of the vonoprazan-containing triple *H. pylori* eradication therapies, using PubMed, EMBASE, and Web of Science. Key words were [“potassium-competitive acid blocker,” “vonoprazan,” or “VPZ”] AND [“*H. pylori* eradication” or “*H. pylori* eradication”]. In addition, we examined the references of the screened articles to identify additional studies. All studies published in English were selected, whereas studies are randomized trial and retrospective observational studies. As the first step of study selection, we excluded irrelevant articles by examining the titles and abstracts of the papers. Next, we screened the full-text of all selected studies. The inclusion criteria were (1) patients: *H. pylori-*positive patients, (2) eradication therapy: vonoprazan-containing triple therapies (first-, second-, and third-line treatments, and in patients with penicillin allergies), and (3) outcome: eradication rate. The exclusion criteria were (1) non-English language and (2) no detail information, such as sample number.

### Efficacy of First-Line Vonoprazan-Containing Eradication Therapy

In recent years, several studies have compared eradication rates between VPZ-containing and PPI-containing triple therapies across centers in Japan. As shown in Table 2, up until September 2018, 23 reports had investigated the efficacy of first-line VPZ-containing therapy (21 reports for VPZ/AMX/CLR and 2 reports for VPZ/MNZ/CLR) (Matsumoto et al., 2016; Murakami et al., 2016; Noda et al., 2016; Shichijo et al., 2016; Shinozaki et al., 2016, 2018; Suzuki et al., 2016; Tsujimae et al., 2016; Yamada et al., 2016; Kajihara et al., 2017; Katayama et al., 2017; Maruyama et al., 2017; Nishizawa et al., 2017; Ono et al., 2017; Sakurai et al., 2017; Sue et al., 2017a,b, 2018a; Sugimoto et al., 2017; Tanabe et al., 2017, 2018; Mori et al., 2018; Ozaki et al., 2018), and 19 reports had compared the efficacy between VPZ-containing therapy and PPI-containing therapy, including 3 randomized control trials (Murakami et al., 2016; Maruyama et al., 2017; Sue et al., 2018a) and 16 non-randomized retrospective cohort trials (Matsumoto et al., 2016; Noda et al., 2016; Shichijo et al., 2016; Shinozaki et al., 2016; Suzuki et al., 2016; Tsujimae et al., 2016; Yamada et al., 2016; Kajihara et al., 2017; Nishizawa et al., 2017; Ono et al., 2017; Sakurai et al., 2017; Sue et al., 2017a,b; Mori et al., 2018; Ozaki et al., 2018; Tanabe et al., 2018).

**Table 2:** Summary of previous studies for the investigation of the efficacy of first-line eradication therapy for *H. pylori* infection.

First author [ref. no.]	Year	Method		VPZ-containing eradication regimen			PPI-containing eradication regimen		
				Number	Regimen	Eradication rate	Number	Regimen	Eradication rate
Murakami (Murakami et al., 2016)	2016	RCT	ITT	329	VPZ: 20 mg bid AMX: 750 mg bid CLR: 200 or 400 mg bid	90.9%	321	LPZ: 30 mg bid AMX: 750 mg bid CLR: 200 or 400 mg bid	75.1%
Suzuki (Suzuki et al., 2016)	2016	RST	ITT	181	VPZ: 20 mg bid AMX: 750 mg bid CLR: 200 or 400 mg bid	89.0%	480	LPZ: 30 mg bid or RPZ: 20 mg bid AMX: 750 mg bid CLR: 200 mg bid	74.2%
Shinozaki (Shinozaki et al., 2016)	2016	RST	ITT	117	VPZ: 20 mg bid AMX: 750 mg bid CLR: 200 or 400 mg bid	82.9%	436	LPZ: 30 mg bid, RPZ: 10 mg bid or EPZ: 20 mg bid AMX: 750 mg bid CLR: 200 mg bid	73.9%
Shichijo (Shichijo et al., 2016)	2016	RST	ITT	422	VPZ: 20 mg bid AMX: 750 mg bid CLR: 200 or 400 mg bid	87.2%	2293	LPZ: 30 mg bid, RPZ: 10 mg bid or EPZ: 20 mg bid AMX: 750 mg bid CLR: 200 or 400 mg bid	72.4%
Noda (Noda et al., 2016)	2016	RST		146	VPZ: 20 mg bid AMX: 750 mg bid CLR: 400 mg bid	89.7%	1305	OPZ: 20 mg bid, LPZ: 30 mg bid, RPZ: 10 mg bid or EPZ: 20 mg bid AMX: 750 mg bid CLR: 200 or 400 mg bid	73.9%
Matsumoto (Matsumoto et al., 2016)	2016	RST	ITT	125	VPZ: 20 mg bid AMX: 750 mg bid CLR: 200 mg bid	89.6%	295	LPZ: 30 mg bid, RPZ: 10 mg bid or EPZ: 20 mg bid AMX: 750 mg bid CLR: 200 or 400 mg bid	71.9%
Yamada (Yamada et al., 2016)	2016	RST	ITT	335	VPZ: 20 mg bid AMX: 750 mg bid CLR: 200 mg bid	85.7%	1720	LPZ: 30 mg bid, RPZ: 10 mg bid or EPZ: 20 mg bid AMX: 750 mg bid CLR: 200 mg bid	73.2%
Tsujimae (Tsujimae et al., 2016)	2016	RST	ITT	443	VPZ: 20 mg bid AMX: 750 mg bid CLR: 200 mg bid	84.6%	431	EPZ: 20 mg bid AMX: 750 mg bid CLR: 200 mg bid	79.1%
Katayama (Katayama et al., 2017)	2017	RST	ITT	258	VPZ: 20 mg bid AMX: 750 mg bid CLR: 200 mg bid	90.6%			
Kajihara (Kajihara et al., 2017)	2017	RST	ITT	111	VPZ: 20 mg bid AMX: 750 mg bid CLR: 400 mg bid	94.6%	98	RPZ: 10 mg bid AMX: 750 mg bid CLR: 200 or 400 mg bid	86.7%
Ono (Ono et al., 2017)	2017	RST	ITT	13	VPZ: 20 mg bid MNZ: 250 mg bid CLR: 200 mg bid	92.3%	10	LPZ: 30 mg bid or RPZ: 10 mg bid MNZ: 250 mg bid CLR: 200 mg bid	50.0%
				14	VPZ: 20 mg bid MNZ: 250 mg bid STFX: 100 mg bid	92.9%	20	LPZ: 30 mg bid or RPZ: 10 mg bid MNZ: 250 mg bid STFX: 100 mg bid	100%
Sakurai (Sakurai et al., 2017)	2017	RST	ITT	546	VPZ: 20 mg bid AMX: 750 mg bid CLR: 200 mg bid	87.9%	807	LPZ: 30 mg bid, RPZ: 10 mg bid or EPZ: 20 mg bid	66.9%
								AMX: 750 mg bid CLR: 200 mg bid	
Sugimoto (Sugimoto et al., 2017)	2017	OS	ITT	76	VPZ: 20 mg bid AMX: 750 mg bid CLR: 200 mg bid	82.9%			
Maruyama (Maruyama et al., 2017)	2017	RCT	ITT	72	VPZ: 20 mg bid AMX: 750 mg bid CLR: 200 or 400 mg bid	95.8%	69	LPZ: 30m g bid or RPZ: 20 mg bid AMX: 750 mg bid CLR: 200 or 400 mg bid	69.6%
Sue (Sue et al., 2017a)	2017	RST	ITT	623	VPZ: 20 mg bid AMX: 750 mg bid CLR: 200 or 400 mg bid	84.9%	608	OPZ: 2- mg bid, LPZ: 30 mg bid, RPZ: 10 mg bid or EPZ: 20 mg bid AMX: 750 mg bid CLR: 200 or 400 mg bid	78.8%
Nishizawa (Nishizawa et al., 2017)	2017	RST	ITT	353	VPZ: 20 mg bid AMX: 750 mg bid CLR: 200 or 400 mg bid	62.3%	2173	LPZ: 30 mg bid or RPZ: 10 mg bid AMX: 750 mg bid CLR: 200 or 400 mg bid	47.1%
Tanabe (Tanabe et al., 2017)	2017	OS	ITT	694	VPZ: 20 mg bid AMX: 750 mg bid CLR: 200 or 400 mg bid	82.7%			
Sue (Sue et al., 2017b)	2017	RST	ITT	20	VPZ: 20 mg bid MNZ: 250 mg bid CLR: 200 or 400 mg bid	100%	30	LPZ: 30 mg bid, RPZ: 10 mg bid or EPZ: 20 mg bid MNZ: 250 mg bid CLR: 200 or 400 mg bid	83.3%
Sue (Sue et al., 2018a)	2018	RCT	ITT	55^∗^	VPZ: 20 mg bid AMX: 750 mg bid CLR: 200 or 400 mg bid	87.3%	51^∗^	LPZ: 30 mg bid, RPZ: 10 mg bid or EPZ: 20 mg bid AMX: 750 mg bid CLR: 200 or 400 mg bid	76.5%
				41^∗∗^	VPZ: 20 mg bid AMX: 750 mg bid CLR: 200 or 400 mg bid	82.9%			
Ozaki (Ozaki et al., 2018)	2018	RST	ITT	1688	VPZ: 20 mg bid AMX: 750 mg bid CLR: 200 or 400 mg bid	90.8%	147	EPZ: 20 mg bid or RPZ: 10 mg bid AMX: 750 mg bid CLR: 200 or 400 mg bid	72.8%
Tanabe (Tanabe et al., 2018)	2018	RST	ITT	363	VPZ: 20 mg bid AMX: 750 mg bid CLR: 200 or 400 mg bid	91.5%	780	LPZ: 30 mg bid, RPZ: 10 mg bid or EPZ: 20 mg bid AMX: 750 mg bid CLR: 200 mg bid	79.4%
Mori (Mori et al., 2018)	2018	RST	ITT	308	VPZ: 20 mg bid AMX: 750 mg bid CLR: 200 or 400 mg bid	81.2%	272	LPZ: 30 mg bid AMX: 750 mg bid CLR: 200 or 400 mg bid	77.6%
Shinozaki (Shinozaki et al., 2018)	2018	OS	ITT	174	VPZ: 20 mg bid AMX: 750 mg bid CLR: 200 or 400 mg bid	83.3%			

*All paper to investigate efficacy of first-line vonoprazan-containing eradication therapy up until September 2018 were listed. ^∗^Clarithromycin-sensitive strain, ^∗∗^Clarithromycin-resistant strain. AMX, amoxicillin; bid, twice daily dosing; CLR, clarithromycin; EPZ, esomeprazole; ITT, intention to treat analysis; LPZ, lansoprazole; MNZ, metronidazole; OPZ, omeprazole; PP, per protocol analysis; PPI, proton pump inhibitor; OS, observational study; RCT, randomized control trial; RST, retrospective cohort trial; RPZ, rabeprazole; VPZ, vonoprazan.*

In 2016, a phase III trial of first-line triple therapy in 650 *H. pylori*-positive subjects showed that the first-line eradication rate was 92.6% (95% confidence interval [CI]: 89.2–95.2%) for the VPZ 20 mg/AMX 750 mg/CLR 200 mg or 400 mg regimen compared to 75.9% (95% CI: 70.9–80.5%) for the lansoprazole/AMX/CLR regimen. There was a difference of 16.7% (95% CI: 11.2–22.1%) in favor of VPZ, confirming the non-inferiority of VPZ (*P* < 0.0001) (Murakami et al., 2016). In another randomizes control trial including 141 *H. pylori*-positive patients with VPZ group (VPZ 20 mg, AMX 750 mg, and CLR 200 or 400 mg) or PPI group (rabeprazole 20 mg or lansoprazole 30 mg, AMX 750 mg, and CLR 200 or 400 mg), the eradication rate was significantly higher in VPZ group (95.8 and 95% CI: 88.3–99.1%) than PPI group (69.6 and 95% CI: 57.3–80.1%, *P* < 0.001) in ITT analysis (Maruyama et al., 2017). In a summary of 21 studies investigating the efficacy of the first-line VPZ/CLR/AMX eradication regimen in 7,469 patients who received VPZ-containing therapy and 12,010 patients who received PPI-containing triple therapy, the studies with eradication rate of more than 85% was 61.2% (13/21 studies) in for VPZ-containing therapy and 0% for PPI-containing therapy (Table 2). Jung et al. (2017) reported that the pooled *H. pylori* eradication rate determined by intention-to-treat (ITT) analysis is 88.1% (95% CI: 86.1–89.9%) in the vonoprazan-containing triple therapy and 72.8% (95% CI: 71.0–75.4%) in PPI-containing triple therapy, respectively, as meta-analysis using 10 reports. In addition, the incidence of any adverse events was similar between both regimens (pooled relative risk [95% CI] = 1.02 [0.78–1.34]) (Jung et al., 2017). However, because most of included studies were retrospective observational studies, this review may have a problem with the quality of the studies (Jung et al., 2017). Meta-analysis should avid to mix randomized case-control studies with cohorts and observational studies.

As shown in Table 2, first-line triple VPZ-containing therapies (VPZ/AMX/CLR) therefore show superior efficacy in Japanese individuals in terms of *H. pylori* eradication compared to PPI-containing therapies (Matsumoto et al., 2016; Murakami et al., 2016; Noda et al., 2016; Shichijo et al., 2016; Shinozaki et al., 2016, 2018; Suzuki et al., 2016; Tsujimae et al., 2016; Yamada et al., 2016; Kajihara et al., 2017; Katayama et al., 2017; Maruyama et al., 2017; Nishizawa et al., 2017; Ono et al., 2017; Sakurai et al., 2017; Sue et al., 2017a,b, 2018a; Sugimoto et al., 2017; Tanabe et al., 2017, 2018; Mori et al., 2018; Ozaki et al., 2018). According to a grading system established by Graham et al. (2007) the 69.1–75.0% eradication rate of PPI-containing therapies constitutes an unacceptable grade (grade F). In contrast, the 86.6–91.7% eradication rate of the VPZ-containing therapies reflects an acceptable grade (grade B or C). Although this rate is by no means excellent, it is a positive step for establishing improved treatment methods in the future. These findings suggest that potent acid inhibition using VPZ is a key requirement for a successful therapy, and is effective despite the high rate of CLR-resistance strains in Japanese individuals.

Increasing the duration from 7 to 10–14 days at eradication is known to increase eradication rates by approximately 5% (Calvet et al., 2000; Fuccio et al., 2007). However, given that there is currently no data on the efficacy of eradication regimens on prolonged eradication periods, further studies are needed to clarify the effectiveness of such therapies.

## First-Line Vonoprazan-Containing Eradication Therapy for Patients Infected With Clarithromycin-Resistant Strains

Implementation of PPI-containing therapy without culture testing is accepted in many countries, whereas *H. pylori* is one of infectious diseases, because eradication treatment based on the culture test requires more time and higher costs compared to non-culture empirical treatment. However, because CLR resistance is becoming a global clinical problem for *H. pylori* eradication in many countries, especially in Japan where the CLR has been used for many patients with bacterial infection, eradication therapy that has been susceptibility tested may be an effective option. CLR is a key antimicrobial agent of current first-line triple *H. pylori* eradication therapies, exerting its antimicrobial effects by binding to the bacterial ribosome 50S subunit to inhibit protein synthesis. The minimum inhibitory concentration used to define resistance to CLR is generally higher than 1.0 mg/mL (Adamek et al., 1998). The susceptibility to CLR in most *H. pylori* strains is conferred by a single nucleotide polymorphism at either position 2142 or 2143 (i.e., A2142G and A2143G) in the *H. pylori* 23S rRNA gene and associated with MIC > 64ug/ml. CLR resistance is therefore a potential confounding factor because CLR resistance significantly affects the efficacy of eradication therapy.

Up until September 2018 (PubMed, EMBASE, Web of Science), six reports had compared the efficacy of first-line VPZ-containing therapy between patients infected with CLR-sensitive and -resistant strains, while 4 reports had compared the efficacy of VPZ/AMX/CLR and PPI (omeprazole 20 mg, lansoprazole 30 mg, rabeprazole 10 mg, or esomeprazole 20 mg)/AMX/CLR therapy (Table 3). In a multicenter prospective randomized clinical trial, the ITT analysis of VPZ/AMX/CLR in the patients infected with CLR-sensitive strain were 87.3% (95% CI: 75.5–94.7%) and that of PPI/AMX/CLR were 76.5% (62.5–87.2%), respectively. No significant difference was observed between the VPZ-containing and PPI-containing regimes in terms of the ITT analysis (*P* = 0.21) (Sue et al., 2018a). A meta-analysis of five original articles (Matsumoto et al., 2016; Murakami et al., 2016; Noda et al., 2016; Sue et al., 2017a, 2018a) showed that in patients infected with CLR-sensitive *H. pylori* strains, eradication rates of VPZ- and conventional PPI-containing therapies were similar in two randomized controlled trials (eradication rate: 95.4% [VPZ] and 92.8% [PPI], odds ratio [OR]: 1.63, 95% CI: 0.74–3.61, *P* = 0.225) (Li et al., 2018). However, VPZ-containing therapy was significantly superior to PPI-containing therapy for patients with CLR-resistant strains in both the randomized controlled trials (eradication rate: 82.0% [VPZ] and 40.0% [PPI], OR: 6.83, 95% CI: 3.63–12.86, *P* < 0.0001) (Li et al., 2018). Compared to the efficacy of conventional PPI-based therapy, the risk of VPZ-containing therapy determined using non-randomized controlled trials was greater for *H. pylori*-positive patients infected with CLR-resistant strains (OR: 5.92) than for *H. pylori*-positive patients infected with CLR-sensitive strains (OR: 2.02) (Dong et al., 2017). Based on this evidence, CLR may be overused given that the combination of VPZ and AMX eradicates approximately 80% of *H. pylori* without CLR (Li et al., 2018). However, as an eradication rate of 80% is not satisfactory, additional measures are needed to obtain a higher eradication rate in patients infected with *H. pylori* CLR-resistant strains.

**Table 3:** Summary of previous studies for the investigation of the efficacy of first-line eradication therapy between clarithromycin-sensitive and -resistant strains.

First author [ref. no.]	Year	Method	Clarithromycin-resistance	VPZ-containing eradication regimen			PPI-containing eradication regimen		
				Number	Regimen	Eradication rate	Number	Regimen	Eradication rate
Murakami (Murakami et al., 2016)	2016	RCT	Sensitive	205	VPZ: 20 mg bid AMX: 750 mg bid CLR: 200 or 400 mg bid	97.6%	185	LPZ: 30 mg bid AMX: 750 mg bid CLR: 200 or 400 mg bid	97.3%
			Resistant	100	VPZ: 20 mg bid AMX: 750 mg bid CLR: 200 or 400 mg bid	82.0%	115	LPZ: 30 mg bid AMX: 750 mg bid CLR: 200 or 400 mg bid	40.0%
Noda (Noda et al., 2016)	2016	RST	Sensitive	44	VPZ: 20 mg bid AMX: 750 mg bid CLR: 400 mg bid	100%	25	OPZ: 20 mg bid, LPZ: 30 mg bid, RPZ: 10 mg bid or EPZ: 20 mg bid AMX: 750 mg bid CLR: 200 or 400 mg bid	88.0%
			Resistant	32	VPZ: 20 mg bid AMX: 750 mg bid CLR: 400 mg bid	87.5%	13	OPZ: 20 mg bid, LPZ: 30 mg bid, RPZ: 10 mg bid or EPZ: 20 mg bid AMX: 750 mg bid CLR: 200 or 400 mg bid	53.8%
Matsumoto (Matsumoto et al., 2016)	2016	RST	Sensitive	57	VPZ: 20 mg bid AMX: 750 mg bid CLR: 200 mg bid	100%	212	LPZ: 30 mg bid, RPZ: 10 mg bid or EPZ: 20 mg bid AMX: 750 mg bid CLR: 200 or 400 mg bid	87.8%
			Resistant	46	VPZ: 20 mg bid AMX: 750 mg bid CLR: 200 mg bid	76.1%	97	LPZ: 30 mg bid, RPZ: 10 mg bid or EPZ: 20 mg bid AMX: 750 mg bid CLR: 200 or 400 mg bid	40.2%
Sugimoto (Sugimoto et al., 2017)	2017	OS	Sensitive	19	VPZ: 20 mg bid AMX: 750 mg bid CLR: 200 mg bid	89.5%			
			Resistant	14	VPZ: 20 mg bid AMX: 750 mg bid CLR: 200 mg bid	78.6%			
Sue (Sue et al., 2017a)	2017	RST	Sensitive	180	VPZ: 20 mg bid AMX: 750 mg bid CLR: 200 or 400 mg bid	88.9%			
			Resistant	56	VPZ: 20 mg bid AMX: 750 mg bid CLR: 200 or 400 mg bid	73.2%			
Sue (Sue et al., 2018a)	2018	RCT	Sensitive	55	VPZ: 20 mg bid AMX: 750 mg bid CLR: 200 or 400 mg bid	87.3%	51^∗^	LPZ: 30 mg bid, RPZ: 10 mg bid or EPZ: 20 mg bid AMX: 750 mg bid CLR: 200 or 400 mg bid	76.5%
			Resistant	41	VPZ: 20 mg bid AMX: 750 mg bid CLR: 200 or 400 mg bid	82.9%			

*All paper to investigate efficacy of first-line vonoprazan-containing eradication therapy investigated susceptibility to antimicrobial agents up until September 2018 were listed. AMX, amoxicillin; bid, twice daily dosing; CLR, clarithromycin; EPZ, esomeprazole; ITT, intention to treat analysis; LPZ, lansoprazole; MNZ, metronidazole; OPZ, omeprazole; PP, per protocol analysis; PPI, proton pump inhibitor; OS, observational study; RCT, randomized control trial; RST, retrospective cohort trial; RPZ, rabeprazole; VPZ, vonoprazan.*

## Second-Line Vonoprazan-Containing Eradication Therapy

Characteristics of patients who require second-line therapy in Japan are infection with a CLR-resistant strain, CYP2C19 extensive metabolizer (EM) phenotype, and poor adherence when first-line treatment was performed. The second-line regimen PPI/AMX/MNZ is currently covered by the Japanese National Health Insurance system, and while the prevalence of patients with *H. pylori* infected with MNZ resistance strain in Japan is 5–12%, the success rate of this second-line regimen has remained constant at approximately 90% (Tsujimae et al., 2016; Yamada et al., 2016; Nishizawa et al., 2017; Ono et al., 2017; Sakurai et al., 2017; Sue et al., 2017a). Japan differs from many other countries in its prevalence rate of the *H. pylori* MNZ-resistant strain, and the high eradication rate following second-line treatment containing MNZ is thought to be country-specific.

Up until September 2018 (PubMed, EMBASE, Web of Science), twelve reports have investigated the efficacy of second-line VPZ-containing therapy and 5 reports have retrospectively compared the efficacy between VPZ/AMX/MNZ and PPI/AMX/MNZ therapy (Table 4). However, a randomized controlled trial on the efficacy of VPZ/AMX/MNZ as a second-line regimen has not been conducted. Murakami et al. (2016) reported that the eradication rate of second-line VPZ-containing eradication therapy (VPZ 20 mg/AMX 750 mg/MNZ 250 mg, bid, 7 days) was high (98.0 and 95% CI: 89.4–99.9%, *n* = 50) in a phase III trial, ranging from 71.8 to 98.0% in 12 reports (Table 4; Murakami et al., 2016; Tsujimae et al., 2016; Yamada et al., 2016; Katayama et al., 2017; Nishizawa et al., 2017; Sakurai et al., 2017; Sue et al., 2017a; Sugimoto et al., 2017; Tanabe et al., 2017; Ozaki et al., 2018). However, there is no significant difference in eradication rates between PPI and VPZ among comparable trials in real-world clinical settings (Tsujimae et al., 2016; Yamada et al., 2016; Nishizawa et al., 2017; Ono et al., 2017; Sakurai et al., 2017; Sue et al., 2017a). A meta-analysis reported in 2017 showed that the eradication rate of VPZ-containing regimens using ITT analysis was similar to that for PPI-containing regimens (83.4% [VPZ] and 81.2% [PPI], OR: 1.04, 95% CI: 0.77–1.42, *P* = 0.79) (Dong et al., 2017). Per protocol (PP) analysis showed comparable results to the ITT analysis (89.3% vs. 90.1%, *P* = 0.06). In addition, the eradication rate is similar among various gastrointestinal diseases (Yamada et al., 2016). The above findings suggest that there is no advantage of using VPZ in second-line treatment. This may be because although AMX is an acid-sensitive antimicrobial agent, MNZ is not an acid-sensitive antimicrobial agent, and potent acid inhibition is not required to increase the stability and bioavailability of MNZ. The mechanism of its antimicrobial effect of MNZ is independent of the bacteria’s stationary or growth phase distribution. In addition, failure to eradicate *H. pylori* using the VPZ/AMX/CLR regimen as first-line therapy likely limits the efficacy of VPZ in second-line therapy containing MNZ.

**Table 4:** Summary of previous studies for the investigation of the efficacy of second-line eradication therapy for *H. pylori* infection.

First author [ref. no.]	Year	Method		VPZ-containing eradication regimen			PPI-containing eradication therapy		
				Number	Regimen	Eradication rate	Number	Regimen	Eradication rate
Murakami (Murakami et al., 2016)	2016	RCT	PP	50	VPZ: 20 mg bid AMX: 750 mg bid MNZ: 250 mg bid	98.0			
Inaba (Inaba et al., 2016)	2016	RST	ITT	37	VPZ: 20 mg bid AMX: 750 mg bid CLR: 200 mg bid	70.2%^∗^			
Yamada (Yamada et al., 2016)	2016	RST	ITT	66	VPZ: 20 mg bid AMX: 750 mg bid MNZ: 250 mg bid	89.6%	386	LPZ: 30 mg bid, RPZ: 10 mg bid, EPZ: 20 mg bid AMX: 750 mg bid MNZ: 250 mg bid	89.9%
Tsujimae (Tsujimae et al., 2016)	2016	RST	ITT	46	VPZ: 20 mg bid AMX: 750 mg bid MNZ: 250 mg bid	89.1%	54	EPZ: 20 mg bid AMX: 750 mg bid MNZ: 250 mg bid	83.3%
Katayama (Katayama et al., 2017)	2017	RST	ITT	24	VPZ: 20 mg bid AMX: 750 mg bid MNZ: 250 mg bid	87.0%			
Ono (Ono et al., 2017)	2017	RST	ITT	1	VPZ: 20 mg bid MNZ: 250 mg bid CLR: 200 mg bid	100%	3	LPZ: 30 mg bid or RPZ: 10 mg bid MNZ: 250 mg bid CLR: 200 mg bid	33.3%
				3	VPZ: 20 mg bid MNZ: 250 mg bid STFX: 100 mg bid	66.7%	24	LPZ: 30 mg bid or RPZ: 10 mg bid MNZ: 250 mg bid STFX: 100 mg bid	100%
Sakurai (Sakurai et al., 2017)	2017	RST	ITT	76	VPZ: 20 mg bid AMX: 750 mg bid MNZ: 250 mg bid	96.1%	185	LPZ: 30 mg bid, RPZ: 10 mg bid or EPZ: 20 mg bid AMX: 750 mg bid MNZ: 250 mg bid	89.7%
Sugimoto (Sugimoto et al., 2017)	2017	OS	ITT	29	VPZ: 20 mg bid AMX: 750 mg bid MNZ: 250 mg bid	93.1%			
Sue (Sue et al., 2017a)	2017	RST	ITT	216	VPZ: 20 mg bid AMX: 750 mg bid MNZ: 250 mg bid	80.5%	146	LPZ: 30 mg bid, RPZ: 10 mg bid or EPZ: 20 mg bid AMX: 750 mg bid MNZ: 250 mg bid	81.5%
Nishizawa (Nishizawa et al., 2017)	2017	RST	ITT	85	VPZ: 20 mg bid AMX: 750 mg bid MNZ: 250 mg bid	71.8%	650	LPZ: 30 mg bid or RPZ: 10 mg bid AMX: 750 mg bid MNZ: 250 mg bid	73.7%
Tanabe (Tanabe et al., 2017)	2017	OS	ITT	73	VPZ: 20 mg bid AMX: 750 mg bid MNZ: 250 mg bid	90.4%			
Ozaki (Ozaki et al., 2018)	2018	RST	ITT	94	VPZ: 20 mg bid AMX: 750 mg bid MNZ: 250 mg bid	86.3%			

*All paper to investigate efficacy of second-line vonoprazan-containing eradication therapy up until September 2018 were listed. AMX, amoxicillin; bid, twice daily dosing; CLR, clarithromycin; EPZ, esomeprazole; ITT, intention to treat analysis; LPZ, lansoprazole; MNZ, metronidazole; OPZ, omeprazole; PP, per protocol analysis; PPI, proton pump inhibitor; OS, observational study; RCT, randomized control trial; RST, retrospective cohort trial; RPZ, rabeprazole; VPZ, vonoprazan.*

In general, second-line therapy with VPZ/AMX/MNZ is well tolerated and patients show good compliance. During the second-line eradication phase, the incidence of treatment-emergent adverse events is 4.0–16.0% (Murakami et al., 2016; Nishizawa et al., 2017). Therefore, in Japan, instead of VPZ/AMX/MNZ, the PPI/AMX/MNZ regimen may be recommended as a second-line treatment due to cost-effectiveness and similar efficacy and safety.

## Third-Line Vonoprazan-Containing Eradication Therapy

The bismuth-containing quadruple regimen and/or sequential or concomitant regimens recommended by the Maastricht V/Florence Consensus Report cannot be used as eradication treatment in Japan because bismuth is not an approved medical drug. Further, third-line eradication therapy is not accepted by the Japanese National Health Insurance system. Because the *H. pylori* strain that infects most patients who receive third-line therapy is resistant to CLR and MNZ, the eradication therapy using PPI/AMX/sitafloxacin (STFX) or PPI/MNZ/STFX, where the PPI is selected, is the main third-line regimen in Japan (Murakami et al., 2013; Furuta et al., 2014a; Sugimoto et al., 2015; Mori et al., 2016). STFX is one of a new quinolone antibacterial agent with anticipated efficacy due to its low MIC for *H. pylori*, including for levofloxacin (LVFX)-resistant strains. Interestingly, STFX has antimicrobial effects to inhibit DNA gyrase and topoisomerase IV, enzymes that are involved in bacteria DNA replication, transcription, DNA repair and recombination (Zhanel et al., 2002). In particular, topoisomerase IV, which consists of ParC and ParE subunits, has an essential role in partitioning replicated chromosomes and is more sensitive than DNA gyrase to some quinolones, such as levofloxacin and ciprofloxacin (Appelbaum and Hunter, 2000). STFX can overcome the resistance of *H. pylori* strains with *gyrA* mutations *in vitro*, (Suzuki et al., 2009) and the low rate of STFX-resistant strains of less than 10% is a strong motivator for its use in eradication therapy (Sugimoto et al., 2015; Mori et al., 2016). Indeed, the effectiveness of STFX-containing eradication therapy has been reported in patients receiving third-line treatment (Murakami et al., 2009, 2013; Suzuki et al., 2009; Hirata et al., 2012; Matsuzaki et al., 2012; Furuta et al., 2014a,b).

Sitafloxacin is also an acid-sensitive antimicrobial agent, whose stability and bioavailability is increased by potent acid inhibition. Therefore, a VPZ/STFX-containing *H. pylori* eradication regimen is expected to increase the eradication rate compared with a PPI/STFX-containing eradication regimen. As shown in Table 5, two reports have investigated the efficacy of third-line VPZ-containing therapy (Sugimoto et al., 2017; Sue et al., 2018b). In a randomized case-controlled trial, patients were divided into a VPZ group (VPZ/AMX/STFX bid for 7 days) or a PPI group (PPI standard dose/AMX/STFX bid for 7 days) (Sue et al., 2018b). Although sample number was limited (*n* = 63), ITT analysis showed that the eradication rates were 75.8% (95% CI: 57.7–88.9%) in the VPZ group and 53.3% (95% CI: 34.3–71.7%) in the PPI group, respectively (Sue et al., 2018b). No significant difference in the frequency of adverse events was evident between the VPZ-containing and PPI- containing regimens. Therefore, although an eradication rate of around 80% remains unsatisfactory, the VPZ/AMX/STFX regimen is more effective than PPI/AMX/STFX as a third-line regimen in patients in whom CLR-containing first-line and MNZ-containing second-line therapies failed to eradicate *H. pylori*. However, because, as mentioned above, *H. pylori* was eradicated in 99% or more patients who received both VPZ-containing first- and second-line treatments, the number of *H. pylori*-positive patients requiring third-line therapy is expected to be considerably reduced, to less than 1%.

**Table 5:** Summary of previous studies for the investigation of the efficacy of third-line eradication therapy for *H. pylori* infection.

First author [ref. no.]s	Year	Method		VPZ-containing eradication regimen			PPI-containing eradication regimen		
				Number	Regimen	Eradication rate	Number	Regimen	Eradication rate
Sugimoto (Sugimoto et al., 2017)	2017	OS	ITT	15	VPZ: 20 mg bid AMX: 500 mg qid STFX: 100 mg bid	80.0%			
Sue (Sue et al., 2018b)	2018	RCT	ITT	33	VPZ: 20 mg bid AMX: 750 mg bid STFX: 100 mg bid	75.8%	30	LPZ: 30 mg bid, RPZ: 10 mg bid or EPZ: 20 mg bid AMX: 750 mg bid STFX: 100 mg bid	53.3%

*All paper to investigate efficacy of third-line vonoprazan-containing eradication therapy up until September 2018 were listed. AMX, amoxicillin; bid, twice daily dosing; EPZ, esomeprazole; ITT, intention to treat analysis; LPZ, lansoprazole; MNZ, metronidazole; PPI, proton pump inhibitor; OS, observational study; qid, four times daily dosing; RCT, randomized control trial; RST, retrospective cohort trial; RPZ, rabeprazole; STFX, sitafloxacin; VPZ, vonoprazan.*

## Vonoprazan-Containing Eradication Therapy in Patients With Penicillin Allergies

Amoxicillin is the most effective and popular drug used for *H. pylori* infection. Therefore, most recommended regimens for *H. pylori* infection include AMX as a key antimicrobial agent. However, because AMX-containing regimens cannot be used by patients with penicillin allergies, eradication regimens that do not contain penicillin derivatives or agents with beta-lactam rings are selected for *H. pylori* eradication in these patients in Japan (Harris et al., 1996; Furuta et al., 2014b). The incidence of penicillin allergies is 3–7% in Japan (Muranaka et al., 1973). For patients who are allergic to penicillin, the Maastricht V/Florence Consensus Report recommends selecting a PPI/CLR/MNZ regimen in areas with low rates of CLR resistance and bismuth-based quadruple therapy in areas of high CLR resistance (Malfertheiner et al., 2017). However, the PPI/CLR/MNZ regimen is associated with an unacceptably low eradication rate, from 55 to 64% according to PP analysis, in patients with penicillin allergies in areas with high rates of CLR resistance (Gisbert et al., 2005, 2010). In contrast, STFX-based triple therapy is reportedly effective in patients with penicillin allergies. Therefore, a PPI/STFX/MNZ regimen may be potentially useful in patients with penicillin allergies, especially in areas with high CLR resistance rates when PPI is used (Murakami et al., 2009, 2013; Suzuki et al., 2009; Hirata et al., 2012; Matsuzaki et al., 2012; Furuta et al., 2014a,b).

Two reports have investigated the efficacy of VPZ-containing therapy in patients with penicillin allergies (Table 6). Ono et al. (2017) reported that the eradication rate of VPZ/MNZ/STFX and VPZ/MNZ/CLR was 92.3% (*n* = 17) and 92.9% (*n* = 14), respectively. Sue et al. (2017b) reported that the efficacy of 7-day first-line treatment with VPZ/MNZ/CLR for *H. pylori* eradication was 100% (95% CI: 86.1–100%; *n* = 20). Because the first-line eradication rate of a VPZ-containing regimen in a CLR-resistant population is around 80% (Murakami et al., 2016), VPZ/STFX/MNZ may potentially be effective in patients with penicillin allergies in areas with a high CLR resistance rate, while VPZ/CLR/MNZ may be effective in areas with a low CLR resistance rate.

**Table 6:** Summary of previous studies for the investigation of the efficacy of first-line eradication therapy for patients with penicillin allergies.

First author [ref. no.]	Year	Method		VPZ-containing eradication regimen			PPI-containing eradication regimen		
				Number	Regimen	Eradication rate	Number	Regimen	Eradication rate
Ono (Ono et al., 2017)	2017	RST	ITT	13	VPZ: 20 mg bid MNZ: 250 mg bid CLR: 200 mg bid	92.3%	10	LPZ: 30 mg bid or RPZ: 10 mg bid MNZ: 250 mg bid CLR: 200 mg bid	50.0%
				14	VPZ: 20 mg bid MNZ: 250 mg bid STFX: 100 mg bid	92.9%	20	LPZ: 30 mg bid or RPZ: 10 mg bid MNZ: 250 mg bid STFX: 100 mg bid	100%
Sue (Sue et al., 2017b)	2017	RST	ITT	20	VPZ: 20 mg bid MNZ: 250 mg bid CLR: 200 or 400 mg bid	100%	30	LPZ: 30 mg bid, RPZ: 10 mg bid or EPZ: 20 mg bid MNZ: 250 mg bid CLR: 200 or 400 mg bid	83.3%

*All paper to investigate efficacy of vonoprazan-containing eradication therapy for patients with penicillin allergies up until September 2018 were listed. bid, twice daily dosing; CLR, clarithromycin; EPZ, esomeprazole; ITT, intention to treat analysis; LPZ, lansoprazole; MNZ, metronidazole; PPI, proton pump inhibitor; RST, retrospective cohort trial; RPZ, rabeprazole; STFX, sitafloxacin; VPZ, vonoprazan.*

## Vonoprazan-Containing Eradication Therapy and CYP3A4/5 Genotype

Vonoprazan is primarily metabolized to its inactive form by CYP3A4/5, and partially by CYP2B6, CYP2C19, and CYP2D6 (Yamasaki et al., 2016). Although the association between plasma VPZ levels and *CYP3A5* genotype is obscure, the elimination rate of VPZ and the formation rate of its major metabolites are significantly correlated with enzyme activity of CYP3A4/5 (Yamasaki et al., 2016), suggesting that CYP3A4/5 activity affects the pharmacokinetics of VPZ, and therefore different clinical outcomes for *H. pylori* eradication among patients with different CYP3A4/5 genotypes. We previously reported that in first-line eradication treatment, the eradication rate in *CYP3A5* ^∗^1 carriers was 72.7% (95% CI 54.5–86.7%), which was significantly lower than that in the *CYP3A5*^∗^3/^∗^3 type (90.7 and 95% CI 69.4–94.1%, *P* = 0.039) (Sugimoto et al., 2017). In univariate analysis of this study, carriage of *CYP3A5* ^∗^3/^∗^3 type was a positive predictive factor for outcome of eradication (OR: 3.656, 95% CI 1.014–13.190, *P* = 0.048) (Sugimoto et al., 2017). *CYP3A5* ^∗^3/^∗^3 type may therefore be a significant positive prognostic predictor of successful eradication when VPZ is used in first-line therapy containing CLR and AMX.

## Tailored Eradication Therapy for *H. pylori* Infection Based on Culture Test

Determining the antibiotic susceptibility using either culture or genetic testing or both is useful to increase the eradication rate, particularly in populations with a high rate of infection with drug-resistant strains. Tailored eradication therapies are promising for significantly increasing successful outcomes compared to standard therapies, particularly in areas with a high prevalence of CLR-resistant strains (Furuta et al., 2007; Kawai et al., 2008; Sugimoto et al., 2014; Ferenc et al., 2017; Cho et al., 2018). A tailored treatment regimen based on susceptibility to CLR (PPI/AMX/CLR for patients infected with CLR-sensitive strains and PPI/AMX/MNZ for those with CLR-resistant strains) achieved a 94.3% eradication rate, which is significantly higher than that achieved with standard treatment (71.4%) (Kawai et al., 2008). Currently, no study has examined the efficacy of VPZ-containing tailored eradication therapy based on susceptibility to CLR. Because the eradication rate of VPZ-containing triple regimen in patients infected with CLR -resistant strains is around 80%, tailored eradication is expected to improve the effectiveness of these therapies.

## Potential Benefits or Limitations of Using Vpz in Populations Outside Japan

Japan is the first country to approve the use of VPZ for clinically acid-peptic disorders, all of studies investigated efficacy of VPZ-containing eradication therapy were reported from Japan. The data on the inhibitory effect on acid secretion of VPZ in Westerners is limited. A recent study showed that at steady state on Day 7, VPZ 40 mg once daily displayed sustained acid inhibition during a 24-h and intragastric pH > 4 HTR in 100% of Japanese and 93.2% of British individuals (Jenkins et al., 2015). Therefore, the effect of suppressing acid secretion of VPZ may differ between Japanese and Westerners. However, acid inhibitory effects of VPZ administration have advantage in patients with CYP2C19 EM, refractory genotype to PPI therapy, suggesting that VPZ will be expected to show usability to Western population with high prevalence of CYP2C19 EMs.

## Conclusion

This review focused on the efficacy of VPZ-containing *H. pylori* eradication therapy in relation to intragastric pH. We discussed the factors effecting the therapeutic outcomes of VPZ-containing *H. pylori* eradication therapy and summarized previous findings using first-, second- and third-line therapies. VPZ-containing triple therapy shows high efficacy in terms of *H. pylori* eradication compared to PPI-containing therapy, especially in patients infected with CLR-resistant strains who received VPZ/AMX/CLR as first-line therapy, patients who received VPZ/AMX/STFX as third-line therapy, and patients with penicillin allergies who received VPZ/AMX/STFX. Importantly, however, efficacy is similar between VPZ-containing and PPI-containing eradication therapy in patients infected with CLR-sensitive strains who received VPZ/AMX/CLR as first-line therapy and patients who received VPZ/AMX/MNZ as second-line therapy. We described the potential of a culture test-based and patients’ pharmacogenomics-based tailored treatment for achieving an eradication rate exceeding 95%. No report to investigate direct association with advantage of vonoprazan use and acid inhibition during vonoprazan-containing eradication therapy using a pH monitoring study consists. Although one of factors affecting *H. pylori* eradication is potent acid inhibition during PPI-containing eradication therapy, it is required to clarify whether theoretical advantages of vonoprazan is caused by potent acid inhibition during eradication therapy as same as PPI-containing eradication therapy.

## Author Contributions

All authors listed have made a substantial, direct and intellectual contribution to the work, and approved it for publication.

## Conflict of Interest Statement

The authors declare that the research was conducted in the absence of any commercial or financial relationships that could be construed as a potential conflict of interest.
